# The cross-talk between autophagy and endoplasmic reticulum stress in blood-spinal cord barrier disruption after spinal cord injury

**DOI:** 10.18632/oncotarget.13777

**Published:** 2016-12-02

**Authors:** Yulong Zhou, Yanqing Wu, Yanlong Liu, Zili He, Shuang Zou, Qingqing Wang, Jiawei Li, Zengming Zheng, Jian Chen, Fenzan Wu, Fanhua Gong, Hongyu Zhang, Huazi Xu, Jian Xiao

**Affiliations:** ^1^ Department of Orthopaedics, The Second Affiliated Hospital and Yuying Children's Hospital, Wenzhou Medical University, Wenzhou, Zhejiang, 325035 China; ^2^ Molecular Pharmacology Research Center, School of Pharmaceutical Sciences, Wenzhou Medical University, Wenzhou, Zhejiang, 325035 China; ^3^ The Institute of Life Sciences, Wenzhou University, Wenzhou 325035, China; ^4^ Department of Neurosurgery, Affiliated Cixi People's Hospital, Wenzhou Medical University, Ningbo, 315300, China

**Keywords:** spinal cord injury (SCI), blood-spinal cord barrier (BSCB), endoplasmic reticulum (ER) stress, autophagy

## Abstract

Spinal cord injury induces the disruption of blood-spinal cord barrier and triggers a complex array of tissue responses, including endoplasmic reticulum (ER) stress and autophagy. However, the roles of ER stress and autophagy in blood-spinal cord barrier disruption have not been discussed in acute spinal cord trauma. In the present study, we respectively detected the roles of ER stress and autophagy in blood-spinal cord barrier disruption after spinal cord injury. Besides, we also detected the cross-talking between autophagy and ER stress both *in vivo* and *in vitro*. ER stress inhibitor, 4-phenylbutyric acid, and autophagy inhibitor, chloroquine, were respectively or combinedly administrated in the model of acute spinal cord injury rats. At day 1 after spinal cord injury, blood-spinal cord barrier was disrupted and activation of ER stress and autophagy were involved in the rat model of trauma. Inhibition of ER stress by treating with 4-phenylbutyric acid decreased blood-spinal cord barrier permeability, prevented the loss of tight junction (TJ) proteins and reduced autophagy activation after spinal cord injury. On the contrary, inhibition of autophagy by treating with chloroquine exacerbated blood-spinal cord barrier permeability, promoted the loss of TJ proteins and enhanced ER stress after spinal cord injury. When 4-phenylbutyric acid and chloroquine were combinedly administrated in spinal cord injury rats, chloroquine abolished the blood-spinal cord barrier protective effect of 4-phenylbutyric acid by exacerbating ER stress after spinal cord injury, indicating that the cross-talking between autophagy and ER stress may play a central role on blood-spinal cord barrier integrity in acute spinal cord injury. The present study illustrates that ER stress induced by spinal cord injury plays a detrimental role on blood-spinal cord barrier integrity, on the contrary, autophagy induced by spinal cord injury plays a furthersome role in blood-spinal cord barrier integrity in acute spinal cord injury.

## INTRODUCTION

The blood-spinal cord barrier (BSCB) is a highly specialized endothelial structure of the fully differentiated neurovascular system between the blood circulation and neural tissue features unique characteristics, which plays an important role in the highly precise control of the microenvironment [1–3]. The BSCB is primarily formed by highly specialized endothelial cells (ECs), which forms a tight structural barrier with the presence of well-developed tight junction (TJ) (Claudins and occludin) Below the TJ, in the basal region of lateral plasma membrane there are adherens junctions (AJ) that mediate events such as the adhesion of ECs to each other, and the regulation of paracellular permeability [4]. Spinal cord injury (SCI) is one of central nervous system (CNS) acute trauma, which usually causes a patient lifelong disability [5, 6] and accompanies with the disruption of BSCB [7]. The disruption of BSCB increases the infiltration of macrophages, loss of TJ and AJ proteins and allows the inflammatory cells enter into injured area, resulting in secondary injury of SCI and finally leading to permanent neurological deficits [1, 8–10].

Endoplasmic reticulum (ER) stress plays an important role on a range of neurological disorders, such as neurodegeneration diseases [11], cerebral ischemia and SCI [12, 13]. And specific role of ER stress in SCI has not been completely defined, especially on BSCB. ER stress is involved in regulation of a series of cell processes, including autophagy [14–16], apoptosis and the complicated regulatory network of them. Recent literatures indicate that GRP78 and PERK/eIF2a are involved in autophagy activation in neural cells [14], and autophagy formation is a cellular defense mechanism against ER stress-mediated apoptosis. On the contrary, ER stress inhibitor inhibits the activation of autophagy [17]. Perior research has reported that the expression of Occludin and Claudin-1 were significantly increased by ER stress inducer thapsigargin (TG) that treated retinal pigment epithelial cells *in vitro* [18, 19]. Additionally, the ER stress inhibitor, 4-phenylbutyric acid (4-PBA), ameliorated ER stress-induced downregulation of Claudin-5 in retinal microvascular ECs [20–22]. However, the relationship between ER stress and BSCB integrity still need to be well described or studied.

Autophagy, a lysosome-dependent cellular degradation pathway, is an essential process for the maintenance of cellular homeostasis in the central nervous system under physiological conditions and pathological conditions [23, 24]. Recently, lots of researchers have focused on the role of autophagy on acute injuries, such as traumatic brain injury and SCI [25–27]. Certain studies have shown that induction of autophagy has neuroprotective effects in acute SCI in rats via inhibitting ER stress-induced apoptosis [28],[29]. In addition, inhibition of autophagy with treating with autophagy inhibitor, 3-methyladenine, resulted in excessive ER stress, leading to upregulation of CHOP and caspase-12 [30, 31]. Moreover, the relationship between autophagy and TJ proteins expression has been described in intestinal epithelial TJ barrier, showing that autophagy upregulated the expression of TJ protein (claudin-2) and protected the TJ barrier [32]. In aneurismal subarachnoid hemorrhage (SAH) model, inhibition of autophagy via treating with 3-methyladenine or wortmannin decreased the neurological scores,, aggravated brain water content and BBB permeability, when compared with the SAH animals [33]. Although the clinical and animal experiment have shown a direct link between a defective BSCB and loss of TJ proteins, the role of autophagy on BSCB integrity during acute SCI is not very clearly.

In present study, we investigated the roles of ER stress and autophagy on BSCB disruption after SCI, aiming to provide theoretical basis for building effective therapeutic interventions of BSCB disruption induced by SCI.

## RESULTS

### Acute SCI leads to the activation of ER stress and autophagy

**Figure 1:**
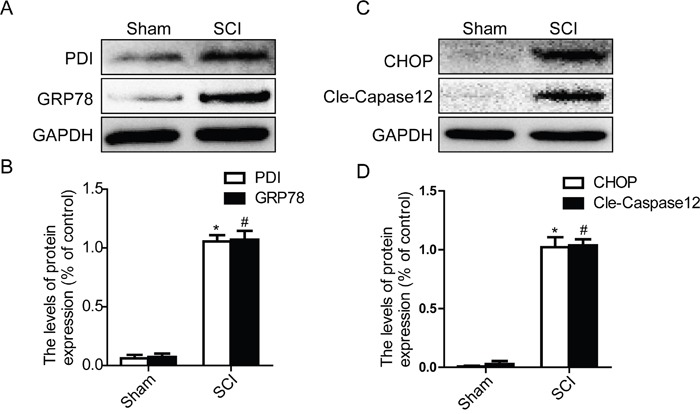
Acute SCI leads to the activation of ER stress at the injury epicenter **A-D.** Representative western blot and quantification data of ER stress markers GRP78, PDI, CHOP, and Cleaved-Caspase12 in the sham and SCI groups. ^*^P< 0.01, ^#^P< 0.01 vs sham group, n≥3.

**Figure 2:**
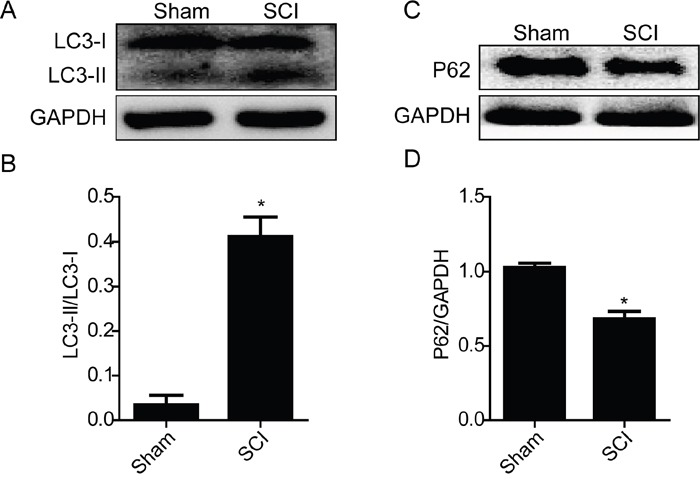
Acute SCI induces activation of autophagy **A** and **B.** Representative western blot and quantification data of autophagy marker LC3-II in the sham and SCI groups. ^*^P< 0.01 vs sham group, n≥3. **C** and **D.** Representative western blots and quantification data of autophagy marker P62 in the sham and SCI groups. ^*^P< 0.05 vs sham group, n≥3.

Prior studies have demonstrated that SCI induces a complex array of tissue responses, including ER stress and autophagy [29, 34]. Here, we test whether ER stress and autophagy were induced at 1 day after SCI. Western blot results showed that the expression levels of GRP78, PDI, CHOP and Cleaved-caspase-12 were significantly increased at early times (1 day) after SCI (Figure 1A, 1B, 1C and 1D). And we also detected the expression level of LC3 protein. The result shown that the ratio of LC3-II/LC3-I was significantly increased in SCI group rats (Figure 2A and 2B). P62, as the biomarker of mature autophagic vesicles [35, 36], was also detected by Western blot. The expression level of P62 was decreased at 1 day after SCI (Figure 2C and 2D). Taken together, consistent with previous study, our results indicate that ER stress and autophagy were involved in the pathomechanism of SCI.

### Acute SCI induces the disruption of BSCB and loss of TJ and AJ proteins

**Figure 3:**
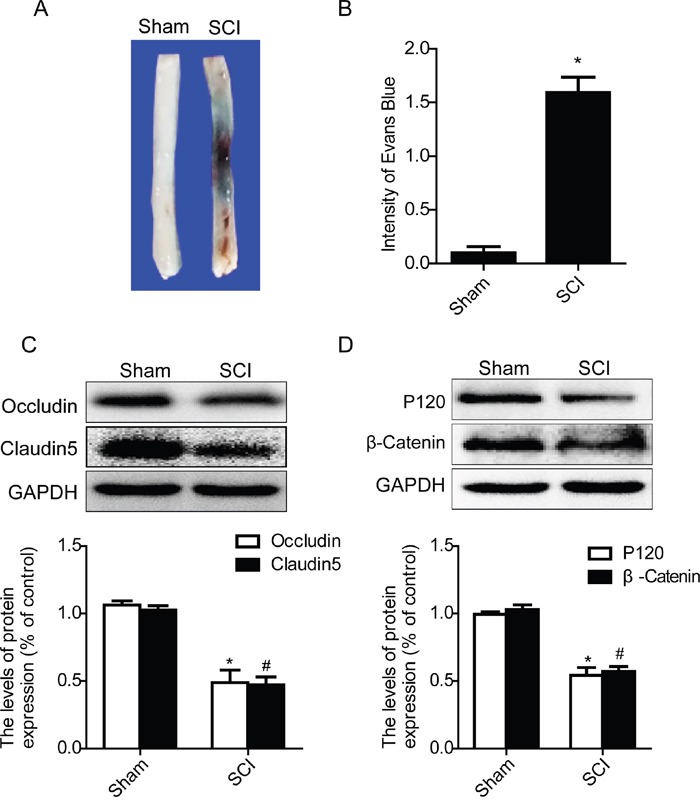
Acute SCI leads to the disruption of BSCB and the loss of TJ and AJ proteins BSCB permeability was measured at 1 d after SCI by using Evan's Blue dye in sham and SCI group. **A** and **B.** Representative whole spinal cords and quantification of BSCB permeability data in each group showing Evan's Blue dye permeabilized into spinal cord. ^*^P< 0.01 vs sham group, n=4. **C** and **D.** Representative western blots and quantification data of TJ proteins (Occludin and Claudin5) and AJ proteins (β-catenin and P120) in the sham and SCI groups. ^*^P< 0.01, ^#^P< 0.01 vs sham group, n≥3.

In present study, we examined the permeability of BSCB at day1 after SCI by Evan's Blue assay, and detected the expression levels of TJ and AJ proteins by western blot. As shown in Figure 3A, the Evan's Blue dye extravasation in SCI group rats was significantly increased when compared with the sham groups, implying BSCB was disruptted. And qualitative analysis also showed that the amount of Evan's Blue dye extravasation at 1 day after SCI was significantly higher in the SCI group than that of sham groups (Figure 3B). Western blot results showed that the expression levels of TJ (Occludin and Claudin5) and AJ (β-catenin and P120) proteins were significantly decreased at 1 day after SCI (Figure 3C and 3D). In a conclusion, consistent with the previous results, these data indicate that SCI induced the disruption of BSCB and decreased the loss of TJ and AJ proteins.

### 4-PBA treatment ameliorates BSCB disruption and reduces autophagy activation after SCI

**Figure 4:**
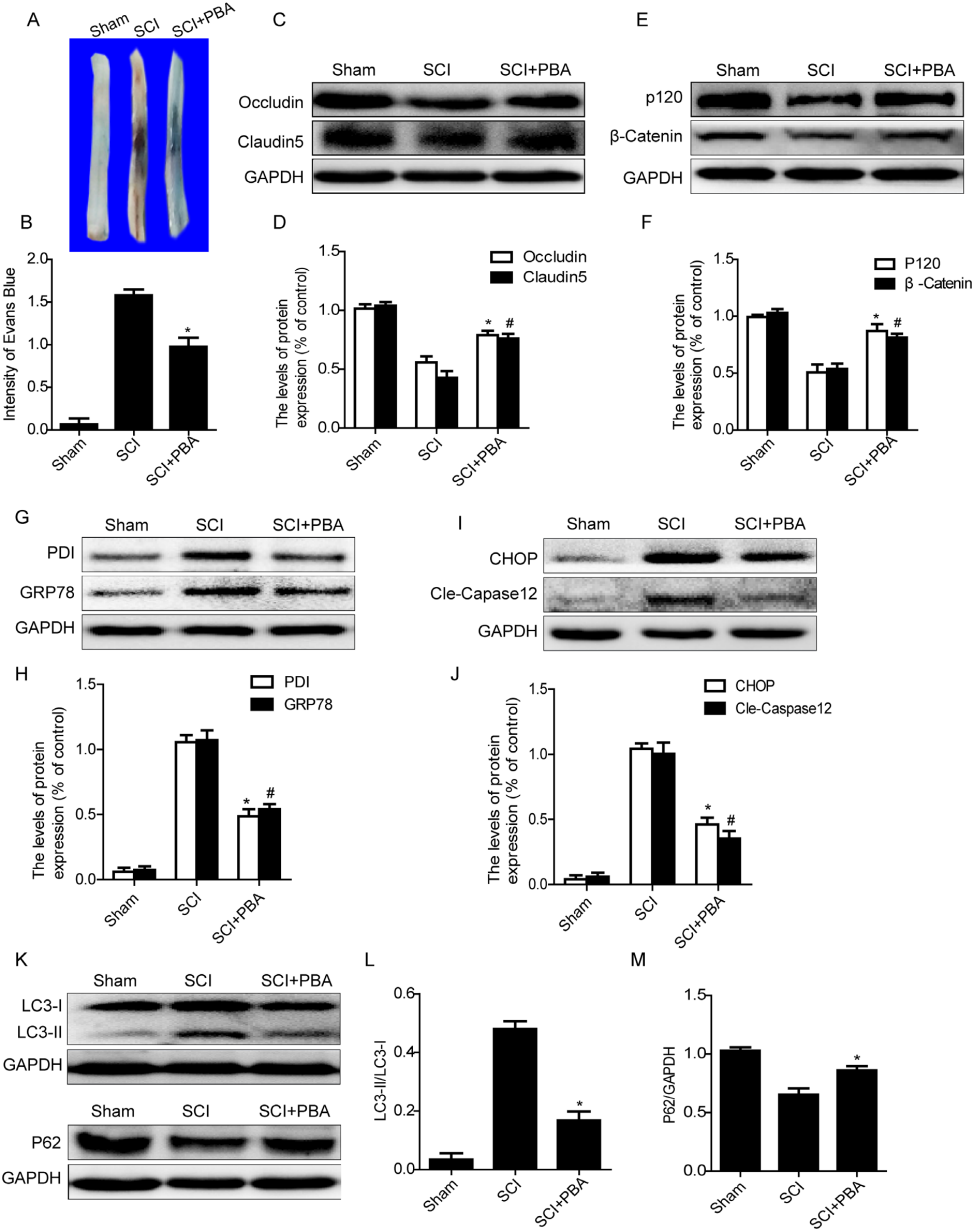
4-PBA treatment ameliorates BSCB disruption and reduces activation of autophagy after SCI **A** and **B.** Representative whole spinal cords and quantification of BSCB permeability data in each group showing Evan's Blue dye permeabilized into spinal cord. ^*^P< 0.01 vs SCI group, n=4. **C-F.** Representative western blots and quantification data of TJ proteins (Occludin and Claudin5) and AJ proteins (β-catenin and P120) in the sham, SCI and SCI+PBA groups. ^*^P< 0.05, ^#^P< 0.05 vs SCI group, n≥3. **G-J.** Representative western blots and quantification data of ER stress markers: GRP78, PDI, CHOP and Cleaved-Caspase12, in the sham, SCI and SCI+PBA groups, ^*^P< 0.01, ^#^P< 0.01 vs SCI group, n≥3. **K-M.** Representative western blots and quantification data of autophagy markers: LC3-II and P62, in the sham, SCI and SCI+PBA groups, ^*^P< 0.05 vs SCI groups, n≥3.

To evaluate the role of ER stress on the disruption of BSCB in SCI, a classical ER stress inhibitor, 4-PBA, was administered into injured rats via intraperitoneal injection after SCI. As shown in Figure 4A and 4B, SCI induced a significant increase in BSCB permeability of Evan's Blue dye compared with the sham groups, which was significantly reduced by treated with 4-PBA. Additionally, as the western blots data shown, 4-PBA treatment increased the expression levels of TJ (Occludin and Claudin5) and AJ (β-catenin and P120) proteins, when compared with that in SCI group(Figure 4C–4F). We also analyzed the expression levels of ER stress associated proteins after 4-PBA treatment. As shown in Figure 4G, 4H, 4I and 4J, the protein levels of GRP78, PDI, CHOP and cleaved-Caspase12 were significantly inhibited in the PBA-treated group as compared with those in the SCI group. These data suggest that 4-PBA treatment inhibited ER stress induced by SCI, which decreased the BSCB permeability and loss of TJ and AJ proteins.

It has been reported that ER stress induced autophagy, which plays important roles in cell survival [15]. Here, we also examined whether inhibition of ER stress by treating with 4-PBA reduces the activation of autophagy. As shown in Figure 4K, 4L and 4M, 4-PBA treatment decreased the expression of LC3-II and upregulated the protein level of P62, when compared with SCI rat groups. Taken together, these results have demonstrated that inhibition of ER stress by treating with 4-PBA effectively inhibited the activation of autophagy after SCI.

### Inhibition of autophagy by chloroquine exacerbates BSCB disruption and enhances ER stress after SCI

**Figure 5:**
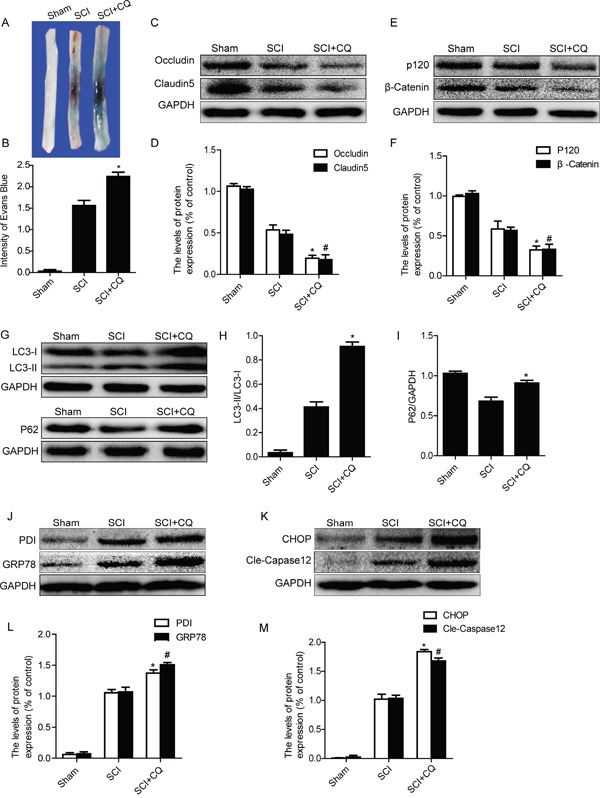
Inhibition of autophagy by chloroquine exacerbates BSCB disruption and ER stress after SCI **A** and **B.** Representative whole spinal cords and quantification of BSCB permeability data in each group showing Evan's Blue dye permeabilized into spinal cord. ^*^P< 0.05 vs SCI groups, n=4. **C-F.** Representative western blots and quantification data of TJ proteins (Occludin and Claudin5) and AJ proteins (β-catenin and P120) in the sham, SCI and SCI+CQ groups. ^*^P< 0.05, ^#^P< 0.05 vs SCI group, n≥3. **G-I.** Representative western blots and quantification data of autophagy markers: LC3-II and P62, in the sham, SCI and SCI+CQ groups, ^*^P<0.05 vs SCI group, n≥3. **J-M.** Representative western blots and quantification data of ER stress markers: GRP78, PDI, CHOP and Cleaved-Caspase12, in the sham, SCI and SCI+CQ groups, ^*^ *P*< 0.01, ^#^ *P*< 0.01 vs SCI group, n≥3.

In order to detect the role of autophagy on BSCB integrality, chloroquine, a classical autophagy inhibitor, was administered into injured rat via intraperitoneal injection after SCI. Evan's Blue assay results showed that, the BSCB permeability of Evan's Blue dye was further increased in chloroquine group as compared with the SCI group (Figure 5A and 5B). We next detected the expression levels of TJ (Occludin and Claudin5) and AJ (β-catenin and P120) proteins by western blots. As shown in Figure 5C–5F, the levels of AJ proteins and TJ proteins were significantly decreased in chloroquine group rats, when compared with those in the SCI group. We also detected the levels of autophagy-associated proteins, such as LC3-II and P62, after treatment with chloroquine by western blot. As shown in Figure 5G and 5H, the expression of LC3-II was further increased in chloroquine group compared with that in the SCI group. On the contrary, the decrease of P62 expression was observed in SCI group, which was partly inhibited in chloroquine group (Figures 5G and 5I). These data indicate that autophagy activation plays a protective role in BSCB integrality after SCI.

Recent studies have reported that inhibition of autophagy enhances ER stress-induced apoptosis in various kinds of cells [30, 31]. We also examined the expression levels of ER stress molecular chaperones (GRP78 and PDI) and ER stress-dependent apoptosis proteins (CHOP and Caspase12) after treating with chloroquine. As Figure 5J–5M shown, the protein levels of GRP78, PDI, CHOP and Caspase12 were significantly increased after treating with chloroquine when compared with those in the SCI group. Taken together, our results indicate that inhibition of autophagy exacerbates BSCB disruption and enhances ER stress after SCI.

### Cross-talking between autophagy and ER stress in the BSCB disruption after acute SCI

**Figure 6:**
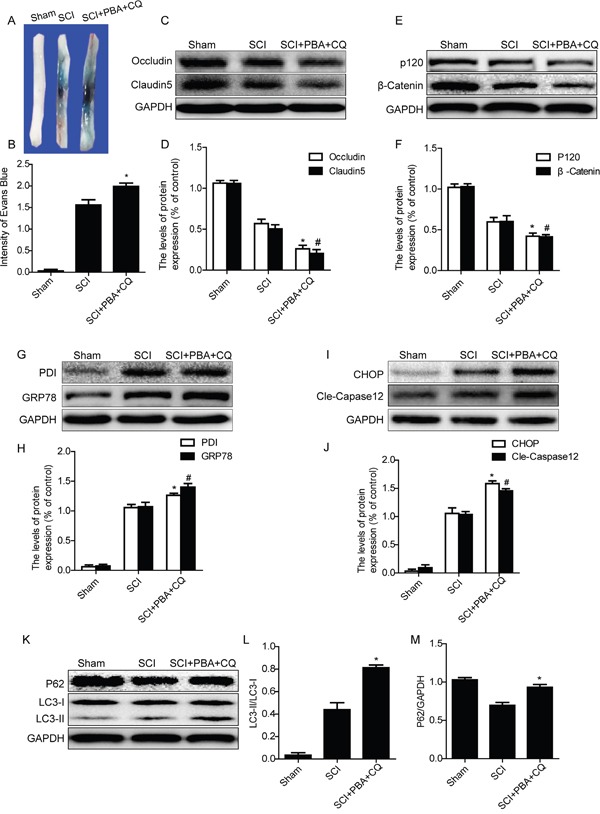
Chloroquine treatment abolishes the BSCB protective effect of PBA by exacerbating ER stress after SCI **A** and **B.** Representative whole spinal cords and quantification of BSCB permeability data in each group showing Evan's Blue dye permeabilized into spinal cord. ^*^P< 0.05 vs SCI groups, n=4. **C-F.** Representative western blots and quantification data of TJ proteins (Occludin and Claudin5) and AJ proteins (β-catenin and P120) in the sham, SCI and SCI+PBA+CQ groups. ^*^P< 0.05, ^#^P< 0.05 vs SCI group, n≥3. **G-J.** Representative western blots and quantification data of ER stress markers: GRP78, PDI, CHOP and Cleaved-Caspase12, in the sham, SCI and SCI+PBA+CQ groups, ^*^P< 0.05, ^#^P< 0.05 vs SCI group, n≥3. **K-M.** Representative western blots and quantification data of autophagy markers: LC3-II and P62, in the sham, SCI and SCI+PBA+CQ groups, ^*^P< 0.05 vs SCI group, n≥3.

In order to determine the role of cross-talking between autophagy and ER stress on the BSCB integrity after SCI, ER stress inhibitor,4-PBA and autophagy inhibitor, chloroquine, were combinedly administrated in injured rats. BSCB permeability was detected by Evan's Blue assay at 1 day after SCI in sham, SCI and SCI+PBA+CQ groups. As shown in Figure 6A and 6B, the BSCB permeability of Evan's Blue dye was further increased in SCI+PBA+CQ group as compared with that in SCI group. Next, we detected the expression of TJ and AJ proteins and found that expression of Occludin, Claudin5, β-catenin and P120 were further decreased in SCI+PBA+CQ group compared with those in SCI group (Figure 6C–6F). These data indicate that the inhibition of autophagy by chloroquine not only abolishes the protective effect of 4-PBA in BSCB and even exacerbates the loss of TJ and AJ proteins after SCI.

We also detected the expression levels of ER stress molecular chaperones (GRP78 and PDI) and ER stress-associated apoptosis proteins (CHOP and Caspase12) after co-treating with PBA and chloroquine in SCI rats. Interestingly, the ER stress inhibition effect of PBA was abolished in SCI+PBA+CQ group rats, evidenced by further increased expression of ER stress associated proteins including GRP78, PDI, CHOP and cleaved-Caspase12, when compared with those in SCI group (Figure 6G–6J). The expression levels of autophagy-associated proteins, such as LC3-II and P62, in SCI+PBA+CQ group were also detected by western blot. As shown in Figure 6K–6M, the expression levels of LC3-II and P62 were further increased in SCI+PBA+CQ group compared with those in SCI group. These results demonstrate that autophagy inhibitor chloroquine abolishes the ER stress inhibition effect of PBA in acute SCI.

### Cross-talking between autophagy and ER stress in TG-treated ECs

**Figure 7:**
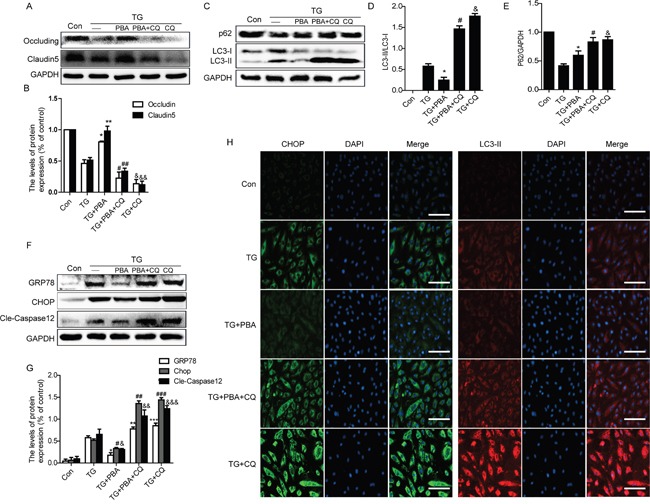
Chloroquine treatment abolishes the role of 4-PBA on ER stress and TJ proteins *in vitro* **A** and **B.** Representative western blots and quantification data of tight junction proteins (Occludin and Claudin5) in each group cells. ^*^P<0.01, ^**^P<0.01, ^#^P<0.05, ^##^P<0.05; ^&^P<0.01, ^&&^P<0.01 vs TG group, n≥3; **C-E.** Representative western blots and quantification data of autophagy markers: LC3-II and P62, in each group cells. ^*^P<0.05, ^#^P<0.01, ^&^P<0.01 vs TG group, n≥3; **F and G.** Representative western blots and quantification data of ER stress markers: GRP78, CHOP and Cleaved-Caspase12 in each group cells. ^*^P<0.01, ^#^P<0.01,^&^P<0.01;^**^P<0.01, ^##^P<0.01, ^&&^P<0.01; ^***^P< 0.01, ^###^P<0.01, ^&&&^P<0.01 vs TG group, n≥3. **H.** Representative images showing Immunofluorescence staining of CHOP (green) and LC3-II (red) in each group cells (original magnification × 200), nuclei are labeled with DAPI (blue) in each group cells. n=4. Scale bar = 50 μm.

To further confirm the role of cross-talking between autophagy and ER stress on the BSCB disruption *in vitro*, thapsigargin (TG) (a classical ER stress inducer), 4-PBA and chloroquine were administrated to HBMVEC. Firstly, we detected the expression of TJ proteins in each group cells by western blot. Consistently with the results *in vivo*, TG-induced downregulation of TJ proteins Occludin and Claudin5 were further enhanced or abolished by chloroquine or PBA, respectively (Figure 7A and 7B). The protective role of 4-PBA on TJ proteins was abolished when HBMVEC co-treated with 4-PBA and chloroquine (Figure 7A and 7B).

Furthermore, compared with the TG-only group cells, the expression levels of LC3-II expression was decreased in TG+PBA group but significantly increased both in TG+PBA+CQ and TG+CQ group cells (Figure 7C and 7D). However, the expression level of P62 was significantly decreased in TG-only group cells, and this decrease was partly inhibited in 4-PBA group (Figure 7C and 7E). Compared with the TG-only group, the expression levels of P62 were significantly increased in TG+PBA+CQ and TG+CQ group. Moreover, we also detect the expression of ER stress-associated protein and observed that 4-PBA treatment ameliorated TG-induced GRP78, CHOP and cleaved-Caspase12 expression, which was abolished by chloroquine treatment (Figure 7F and 7G). Double labelling immunofluorescence of CHOP and LC3-II results were consistent with the western blot results (Figure 7H). These data suggest that the 4-PBA treatment remits the role of ER stress on TJ proteins expression in HBMVEC, which was abolished by treating with chloroquine.

## DISCUSSION

Spinal cord injury (SCI) is a severe health problem, which results in rapid, permanent changes to the structure and function of the microvessels at the cellular level, leading to increase the permeability of BSCB [7, 8]. At 1 day postinjury, the BSCB is significantly disrupted, compared to sham animals, as well as all other sampled time points, indicating that the BSCB is maximally disrupted at 1 day after clip-compression SCI [7]. In present study, we observed that SCI induces BSCB permeability and the loss of TJ and AJ proteins at 1 day postinjury. It is well known that the expression levels and distribution of TJ and AJ proteins are closely related to BSCB integrity [37]. Our results confirmed the role of TJ and AJ proteins on BSCB integrity.

ER stress occurs when misfolded proteins accumulate in ER lumen and cause ER dysfunction [38]. Excessive ER stress may lead to apoptosis by activating CHOP and Cleaved-Caspase12, resulting in secondary injury after SCI [12, 39]. Previous study has reported that following contusive SCI, CHOP deficiency in mice significantly preserves microvascular density and attenuated macrophage infiltration when compared to wild type mice [40]. Consistent with the prior study, our present study has also found that ER stress was significantly induced by SCI and 4-PBA treatment, a classical ER stress inhibitor, ameliorated the effect of SCI on BSCB permeability and the loss of AJ and TJ proteins, which also been found in a TG-treated HBMVEC model *in vitro.* Above data suggest that ER stress plays an adverse role on BSCB integrity after SCI.

Autophagy is a lysosome-dependent essential cellular catabolic pathway that serves to degrade cytoplasmic proteins, protein aggregates and organelles [23, 41]. There are some evidences reported that moderate autophagy protect ECs against cell injury under stressful circumstances [24, 32, 42–45]. In aneurismal subarachnoid hemorrhage (SAH) model, inhibition of autophagy by 3-methyladenine and wortmannin decreased neurological scores and further aggravated brain water content and BBB permeability, when compared with the SAH animals [33]. According to the above studies, the protective potential of autophagy in various ECs damage is evident [46, 47]. Although both clinical data and animal experiment show a direct link between a defective BSCB and the loss of TJ proteins, the role of autophagy in the regulation of the TJ proteins remains unknown, especially in acute SCI. In the current study, our results indicate that autophagy was increased in acute SCI, and inhibition of autophagy by chloroquine increased the BSCB permeability and promoted the loss of TJ and AJ proteins after SCI, suggesting that autophagy activation induced by SCI plays a protective role on BSCB integrity after SCI.

Prior and our studies have shown that ER stress and autophagy activation are both involved in the regulation of BSCB disruption induced by SCI [29, 48, 49]. The cross-talking between ER stress and autophagy during the regulation of BSCB integrity after SCI is still unclear and need further investigation. A recent study indicates that SCI causes lysosomal dysfunction that contributes to autophagy disruption and associated ER-stress-induced neuronal apoptosis [29]. The activity of autophagy by rapamycin is against endoplasmic reticulum stress and inflammation in adipocytes [50]. In addition, inhibition autophagy by chloroquine exacerbated the expression of ER stress markers and co-treatment of chloroquine abolished the anti-ER stress effects of rapamycin [50]. On the contrary, ER stress inhibitor inhibited the activation of autophagy and ER stress-induced autophagy might contribute to the neuroprotective effect of brain ischemic preconditioning [17]. The cross-talking between autophagy and ER stress may play an important role in the recovery of CNS disorders. In present studies, we have focused on the cross-talking between autophagy and ER stress *in vivo* and *in vitro*. We found that 4-PBA treatment significantly reduced the activation of autophagy *in vivo* and *vitro*, however, the inhibition autophagy by chloroquine aggravated the ER stress. Additionally, the protective effect of 4-PBA on BSCB integrity and AJ and TJ proteins was abolished when 4-PBA co-treating with chloroquine. All of above data highlight the regulation of balance between autophagy and ER stress exerts an important role in protecting BSCB integrity after SCI. However, the molecular mechanisms by which autophagy inhibit ER stress-dependent apoptotic pathway remain to be determined.

Although the cross-talk between autophagy and ER stress in SCI has been reported, the mechanism that links the cross-talk between autophagy and ER stress to the regulation of TJ and AJ proteins is currently lacking. In our following study, CHOP and Atg5 knockout rats will be applied to further reveal the role of cross-talking between autophagy and ER stress in BSCB disruption and TJ proteins regulation after acute SCI.

**Figure 8:**
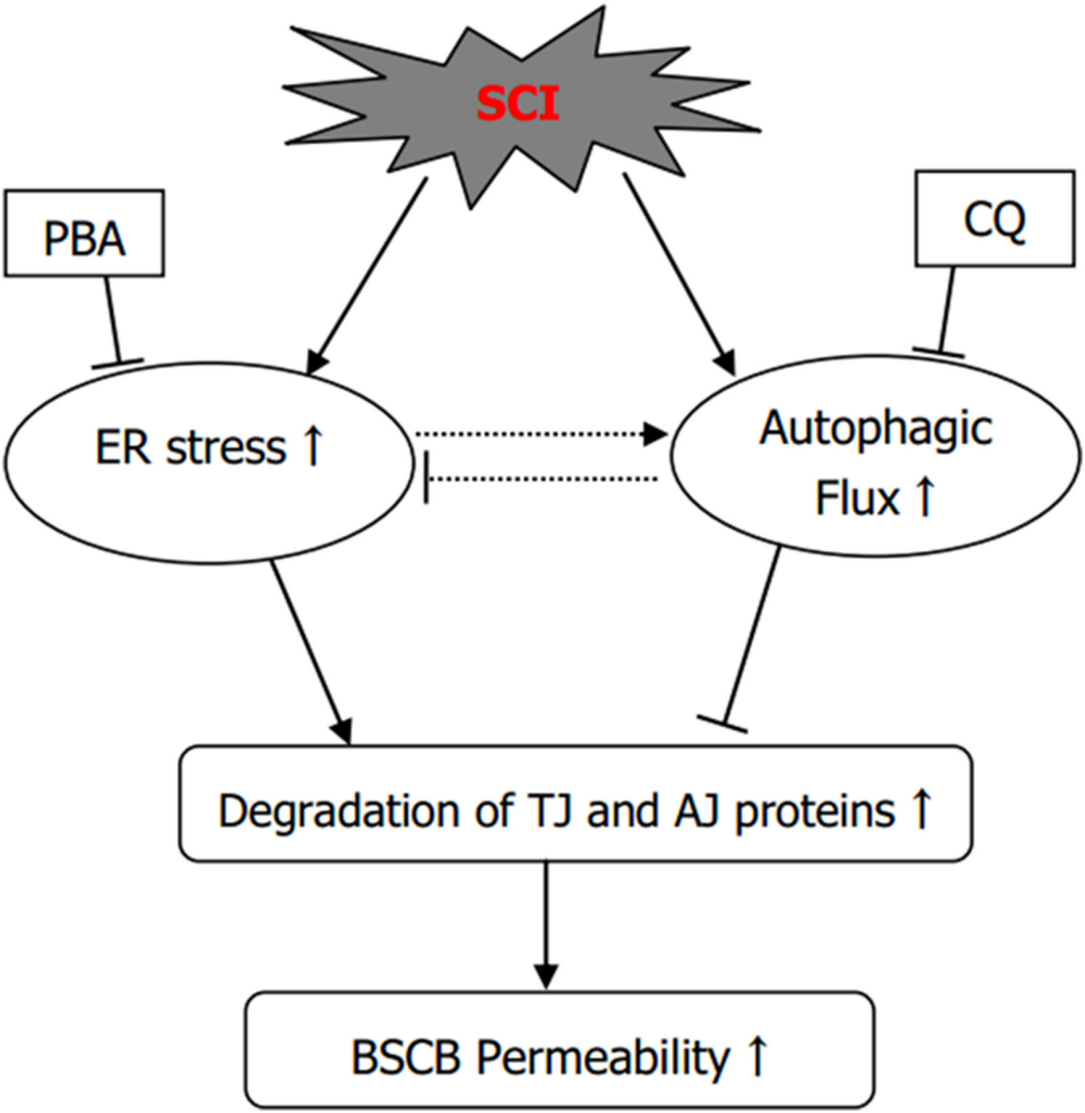
The illustration for the roles of ER stress and autophagy on BSCB disruption after SCI SCI induces ER stress and autophagy, which promotes the degradation of TJ and AJ proteins and finally leading to BSCB disruption. PBA treatment reduces the activation of autophagy and ameliorates BSCB disruption and loss of TJ and AJ proteins. Chloroquine treatment enhances ER stress and aggravates BSCB disruption and loss of TJ and AJ proteins.

In conclusion, the current study respectively examined the roles of ER stress and autophagy in BSCB disruption after SCI. ER stress induced by SCI plays a detrimental role in BSCB integrity and promotes the loss of TJ and AJ proteins after SCI, however, autophagy plays a furthersome role in BSCB integrity and prevents loss of TJ and AJ proteins (Figure 8). And we also partially clarified the role of cross-talking between autophagy and ER stress in BSCB disruption and TJ proteins regulation in acute SCI model, which may provide new knowledge to understand the regulation of BSCB integrity after SCI and establish theoretical basis for building effective therapeutic interventions during SCI recovery.

## MATERIALS AND METHODS

### Animal model of SCI and drug treatment

All experimental procedures were approved by the ethics committee of Wenzhou Medical University and performed in accordance with the Guide for the Care and Use of Laboratory Animals. Adult female Sprague-Dawley (SD) rats (220-250g) were purchased from Animal Center of Chinese Academy of Science, Rats were maintained under temperature controlled environment (23-25°C) with 12 h light/dark cycles and free access to food and tap water. All rats were anaesthetized using 10 % chloralic hydras (3.6 ml/kg) via intraperitoneal injection beforea laminecormy. After the T9 vertebrae of rats were ascertained, all fur and muscle adjacent to the spinous processes were dislodged to expose the vertebral column, and a laminectomy was performed at the level of T9 vertebral level to expose the spinal cord. Then, rats were suffering from a crush injury via compression with a vascular clip (30 g forces, Oscar, China) for 2 minutes. The sham group rats received the same surgical procedure but without a compression injury. The incision sites were then closed in layers and a topical antibiotic (cefazolin sodium salt, 50 mg/kg, i.p.) was applied. 4-PBA (Sigma-Aldrich, St. Louis, MO, USA) was dissolved to a stock solution of 100 mg/mL in 100% DMSO and immediately administered by i.p.injection (100 mg/kg) after SCI. Chloroquine (Sigma-Aldrich, St. Louis, MO, USA) was dissolved to a stock solution of 150 mg/mL in PBS and immediately administered by i.p. injection (50 mg/kg) together with 4-PBA or not. Equal doses of PBS and DMSO were administered to vehicle treated rats. Regarding postoperative care, manual urinary bladder emptying was performed twice daily until bladder function returned. Following completion of the trial, the rats were euthanized via overdose of chloral hydrate.

### Cell culture

Primary cultures of Human Brain Microvascular Endothelial Cells (HBMVEC) were purchased from ScienCell Research Laboratories (ScienCell Research Laboratories, San Diego, CA, USA) and maintained at 37°C in a humidified atmosphere containing 5% CO_2_ (v/v). HBMEC were cultured in Endothelial Cell Medium (ECM). To further assess the effects of ER stress activation and autophagy after SCI, cells were treated with TG (10 μM), 4-PBA (1 mM) and PBA compound with chloroquine (100 μM) or chloroquine alone. All experiments were performed in triplicate.

### Measurement of BSCB disruption

According to previous reports, the integrity of BSCB was evaluated with Evans blue dye extravasation at 1 day after SCI [51, 52]. At 1 day after SCI, 4 ml/kg 2% Evan's Blue dye solution (Sigma-Aldrich) was injected intravenously into animal's tail vein. Three hours later, animals were anaesthetized and killed by intra-cardiac perfusion with saline. 1cm T9 spinal cord surrounding the injury site was extracted and weighed, and snap-frozen in dry ice and then homogenized in 50% trichloroacetic acid solution. Samples (400 mg) were then homogenized in 400 μL of N,N-dimethylformamide (DMF) and incubated at 70°C for 72 h. Then, samples were centrifuged at 18,000 rpm for 20 min twice. The supernatant was collected, and aliquoted (200 μl) into 96-well glass plate,.its fluorescence was quantified using a spectrophotometer at an excitation wavelength of 620 nm and emission wavelength of 680 nm. Dye in samples was determined as micrograms per gram of tissue from a standard curve of EB in DMF.

### Western blot analysis

For protein analysis *in vivo*, a spinal cord segments (0.5 cm length) at day 1 after SCI was dissected and rapidly stored at −80°C for western blotting. Total protein from the spinal cord tissues and HBMEC were purified using lysis buffer with protease inhibitors. Tissues or cells were incubated for 20 min at 4°C after homogenates, then centrifuged at 12,000 rpm for 15 min at 4°C. The protein concentration was determined using the BCA protein assay kit (Pierce, Rockford, IL, USA). The equivalent of 75 μg (*in vivo*) or 50 μg (*in vitro*) of proteins were separated by SDS-PAGE gel, then transferred onto a PVDF membrane (Bio-Rad Laboratories). The membrane was blocked with 5% non-fat milk in TBST for 90 min at room temperature and then incubated overnight at 4°C with primary antibodies as follows: Occludin(Santa Cruz, 1:800), Claudin5 (Santa Cruz, 1:800), P120 (Abcam, 1:1000), β-Catenin (Abcam, 1:1000), CHOP (Santa Cruz, 1:300), GRP78 (Santa Cruz, 1:300), PDI (Abcam, 1:1000), Cleaved-caspase12 (Abcam, 1:1000), LC-3II (Cell Signaling Technologies, 1:1000), P62 (Cell Signaling Technologies, 1:1000). After being washed in TBST, the membrane was incubated with goat anti-rabbit or goat ant-mouse secondary antibody for 1 h at room temperature, and bands were detected using an enhanced chemiluminescence (ECL) kit. Band intensity was quantified using Image Lab 3.0 software (Bio-Rad). Experiments were repeated three times.

### Immunofluorescence staining

The HBMEC grown to confluence on fibronectin-coated coverslips were subjected to the indicated treatments. Cells were washed in PBS, fixed in 4% paraformaldehyde for 30 min, and blocked for 30 min at 37°C with 5% BSA. Then the HBMEC were incubated with antibodies against LC3-II (1:400), CHOP (1:100) at 4°C for overnight. Cells were rinsed three times in PBS after primary antibody incubation and then were sequentially incubated with Alexa Fluor 594/647 donkey anti-mouse/rabbit or Alexa-Fluor 488/594 donkey anti-rabbit/mouse secondary antibody (Abcam, 1:500) for 2h at room temperature. After that, the cells were stained with DAPI for 10 min at room temperature. Then the cells were mounted on slides and images of fluorescence were captured using a confocal microscope (Nikon, Japan).

### Statistical analysis

Data are presented as the mean ± SEM. Statistical significance between two experimental groups was analyzed using Student's t -test. When more than two groups were compared, one-way ANOVA and Dunnett's post hoc test were used. P<0.05 was considered to indicate statistical significance.
